# The nucleoid protein HU positively regulates the expression of type VI secretion systems in *Enterobacter cloacae*

**DOI:** 10.1128/msphere.00060-24

**Published:** 2024-04-22

**Authors:** Gabriela Hernández-Martínez, Miguel A. Ares, Roberto Rosales-Reyes, Jorge Soria-Bustos, Jorge Antonio Yañez-Santos, María L. Cedillo, Jorge A. Girón, Ygnacio Martínez-Laguna, Fenfei Leng, J. Antonio Ibarra, Miguel A. De la Cruz

**Affiliations:** 1 Unidad de Investigación Médica en Enfermedades Infecciosas y Parasitarias, Hospital de Pediatría, Centro Médico Nacional Siglo XXI, Instituto Mexicano del Seguro Social, Mexico City, Mexico; 2 Escuela Nacional de Ciencias Biológicas, Instituto Politécnico Nacional, Mexico City, Mexico; 3 Unidad de Medicina Experimental de la Facultad de Medicina, Universidad Autónoma de México, Mexico City, Mexico; 4 Pathogen and Microbiome Division, Translational Genomics Research Institute (TGen) North, Flagstaff, Arizona, USA; 5 Instituto de Ciencias de la Salud, Universidad Autónoma del Estado de Hidalgo, Pachuca, Hidalgo, Mexico; 6 Centro de Detección Biomolecular, Benemérita Universidad Autónoma de Puebla, Puebla, Mexico; 7 Centro de Investigación en Ciencias Microbiológicas, Benemérita Universidad Autónoma de Puebla, Puebla, Mexico; 8 Biomolecular Sciences Institute and Department of Chemistry and Biochemistry, Florida International University, Miami, Florida, USA; 9 Facultad de Medicina, Benemérita Universidad Autónoma de Puebla, Puebla, Mexico; University of Kentucky College of Medicine, Lexington, Kentucky, USA

**Keywords:** *E. cloacae*, T6SS, HU, nucleoid protein

## Abstract

*Enterobacter cloacae* is an emerging pathogen isolated in healthcare-associated infections. A major virulence factor of this bacterium is the type VI secretion system (T6SS). The genome of *E. cloacae* harbors two T6SS gene clusters (T6SS-1 and T6SS-2), and the functional characterization of both systems showed that these two T6SSs are not expressed under the same conditions. Here, we report that the major histone-like protein HU positively regulates the expression of both T6SSs and, therefore, the function that each T6SS exerts in *E. cloacae*. Single deletions of the genes encoding the HU subunits (*hupA* and *hupB*) decreased mRNA levels of both T6SS. In contrast, the *hupA hupB* double mutant dramatically affected the T6SS expression, diminishing its transcription. The direct binding of HU to the promoter regions of T6SS-1 and T6SS-2 was confirmed by electrophoretic mobility shift assay. In addition, single and double mutations in the *hup* genes affected the ability of inter-bacterial killing, biofilm formation, adherence to epithelial cells, and intestinal colonization, but these phenotypes were restored when such mutants were *trans*-complemented. Our data broaden our understanding of the regulation of HU-mediated T6SS in these pathogenic bacteria.

**IMPORTANCE:**

T6SS is a nanomachine that functions as a weapon of bacterial destruction crucial for successful colonization in a specific niche. *Enterobacter cloacae* expresses two T6SSs required for bacterial competition, adherence, biofilm formation, and intestinal colonization. Expression of T6SS genes in pathogenic bacteria is controlled by multiple regulatory systems, including two-component systems, global regulators, and nucleoid proteins. Here, we reported that the HU nucleoid protein directly activates both T6SSs in *E. cloacae*, affecting the T6SS-related phenotypes. Our data describe HU as a new regulator involved in the transcriptional regulation of T6SS and its impact on *E. cloacae* pathogenesis.

## INTRODUCTION

*Enterobacter cloacae* are saprophytic environmental bacteria and are part of the human gut microbiota (1). This Gram-negative bacterium is an important opportunistic pathogen in healthcare-associated infections (HAIs) in patients hospitalized in the intensive care unit. These infections encompass the lower respiratory tract, urinary tract, and meninges (2). *E. cloacae* virulence factors associated with biofilm formation and cytotoxic activity on eukaryotic cells have been described (3–6).

One of the virulence factors recently studied in *E. cloacae* is the type VI secretion system (T6SS). This nanomachine was discovered in 2006 as a complex macromolecular apparatus found in more than 25% of sequenced genomes of Gram-negative bacteria (7–9). At the molecular level, the T6SS is assembled via interactions between 13 canonical proteins, called Tss proteins. Several of these proteins share structural homologies with components of the T4 bacteriophage contractile tail, such as the major tail tube protein (Hcp), the cell puncturing device (VgrG), the sheath (TssB-C), and at least one component of the baseplate (TssE) (8, 10, 11). The genomic analysis of *E. cloacae* ATCC 13047 revealed that it possesses two T6SS clusters (2), which our group named T6SS-1 and T6SS-2 (12). The functional characterization of both systems showed that these two T6SSs are not expressed under the same environmental conditions (12). However, the regulation of the expression of these T6SSs in *E. cloacae* has not been studied.

The biogenesis and function of T6SS are paramount as it is energetically costly to bacterial cells, necessitating tight control over its gene expression to adapt its expression and assembly to changing environmental conditions. A plethora of environment modulators and regulatory systems have been reported in bacteria to control the transcription of T6SSs either directly or indirectly. Furthermore, beyond the transcription mechanisms, several T6SSs are post-translationally activated by a threonine phosphorylation pathway in response to cell damage or envelope stress.

Nucleoid-associated proteins (NAPs) are abundant proteins in bacterial cells involved in many important cellular processes such as genome architecture, physiology, metabolism, stress response, and virulence (13–15). One of the first NAPs to be described in detail was HU (*H*istone-like protein from *Escherichia coli* strain *U*93) (16). In bacteria that belong to the *Enterobacteriaceae* and *Vibrionaceae* families (13, 17), HU, the histone-like protein most abundant on the bacterial nucleoid, is a heterodimer formed of two subunits, HupA (HUα) and HupB (HUβ) (18). HU protein has three naturally occurring forms: the HUα_2_ and HUβ_2_ homodimers and the HUαβ heterodimer. The heterodimer binds DNA in a non-specific manner, contributing to DNA flexibility by bending the duplex, which is essential for the structural integrity of the chromosome (19–24) and regulation of virulence factors in several enterobacterial species (25–28). However, two genome analyses identified HU protein-binding motifs revealing A/T- and T/G-rich sequences in *E. coli* and *Francisella tularensis*, respectively (29, 30).

Currently, the role of the HU protein as a global regulator in the biology of *E. cloacae* is unknown. Nevertheless, in its close relative, *E. coli* HU regulates the expression of 353 genes that respond to anaerobiosis, acid stress, high osmolarity, and SOS response (17, 31).

In this study, we demonstrated that HU positively regulates the T6SS gene expression in *E. cloacae* by binding directly to their promoter regions in *E. cloacae*. Deleting either *hupA* or *hupB* genes results in a significantly reduced transcription of gene clusters encoding the T6SS-1 and T6SS-2. Consistently, virulence phenotypes such as inter-bacterial competition, biofilm formation, and adherence to epithelial cells were severely affected in the *hupA* or *hupB* mutants but restored when *hupA* or *hupB* genes were overexpressed in *trans* from plasmids. To our knowledge, this is the first study that addresses the transcriptional regulation of the T6SS gene cluster, in which HU acts as a direct activator, turning on the T6SS-associated phenotypes.

## RESULTS

### The absence of HU affects the *E. cloacae* bacterial growth

**Fig 1:**
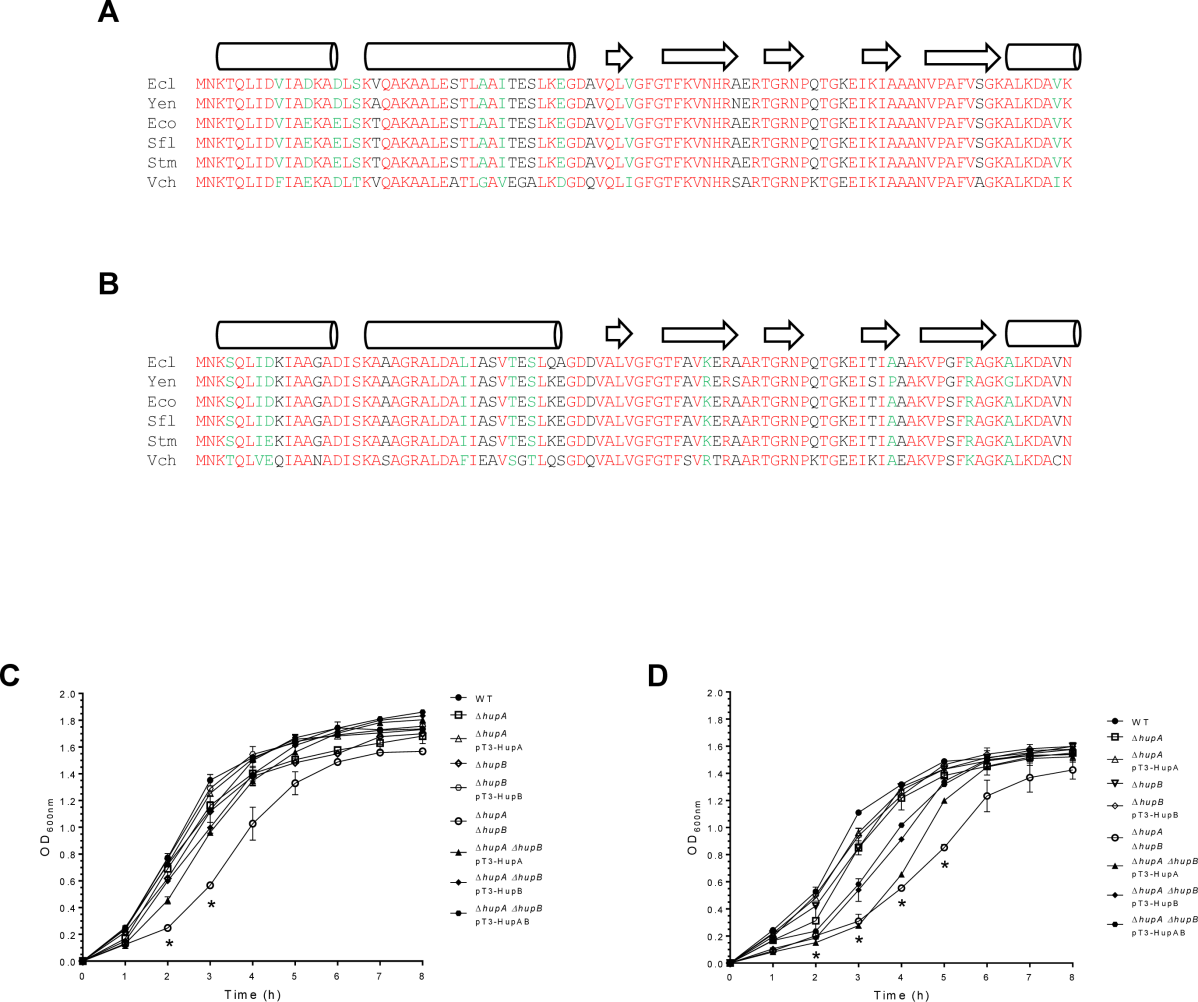
HU protein is required for the *E. cloacae* growth. Alignment of the amino acids sequence of HupA (**A**) and HupB (**B**) subunits of different bacteria: *E. cloacae* strain ATCC 13047 (ADF59828.1/ADF60759.1, Ecl), *E. coli* strain MG1655 (NP_418428.1/NP_414974.1, Eco), *S. flexneri* 2 a strain 301 (NP_709794.1/NP_706334.1, Sfl), *S*. Typhimurium strain LT2 (NP_463039.1/NP_459447.1, Stm), *Y. enterocolitica* (WP_005166072.1/WP_004392663.1, Yen), and *V. cholerae* O1 biovar El Tor strain N16961 (Q9KV83.1/Q9KQS9.1, Vch). This analysis was performed using the Clustal Omega software (https://www.ebi.ac.uk/Tools/msa/clustalo/). Red and green colors were used to mark identical and similar residues, respectively. Prediction of secondary structures such as α-helices (cylinders) and β-sheets (arrows) are depicted, and it was performed using the SWISS-MODEL software (https://www.expasy.org/resources/swiss-model). Growth curves of WT, *hup* isogenic mutants and complemented mutant strains grown in TSB (**C**) and DMEM (**D**) at 37°C and 200 rpm. These graphs represent the mean of three independent experiments performed in triplicate with standard deviations. Statistically significant in relation to the WT bacteria; *: *P* < 0.05.

To begin this study, we analyzed homolog sequences to both HU subunits of *E. cloacae* in comparison to the enteric bacteria *E. coli*, *Shigella flexneri*, *Yersinia enterocolitica*, *Vibrio cholerae*, and *Salmonella* Typhimurium. Identical and similar amino acid residues were identified in HupA (HUα; Fig. 1A) and HupB (HUβ; Fig. 1B) subunits when the polypeptide sequences were aligned. *E. cloacae* HU protein showed 97%, 97%, 96%, 95%, and 77% identity percentages to HU protein *E. coli*, *S. flexneri*, *S*. Typhimurium, *Y. enterocolitica*, and *V. cholerae*, respectively. The prediction of secondary structures showed the same number of α-helices and β-sheets for each HU subunit, supporting the high identity between them.

Next, single and double mutants in both HU subunits were generated in *E. cloacae* ATCC 13047, and those strains were evaluated in their growth with respect to the wild-type (WT) strain using tryptic soy broth (TSB) and Dulbecco’s modified Eagle’s medium (DMEM), two synthetic media previously used in our group, which activate the T6SS-1 and T6SS-2, respectively (12). Independent of the culture medium, only the Δ*hupA*Δ*hupB* double mutant showed a significant effect (*P* < 0.05) on bacterial growth, and such defect was restored when both subunits were co-expressed in *trans* (Fig. 1C and D). In this sense, the *trans*-complementation of Δ*hupA*Δ*hupB* with both HU single subunits expressed in plasmids revealed that only the HUβ subunit was able to restore the *E. cloacae* growth when *hupB* is overexpressed.

### HU positively regulates both T6SS clusters in *E. cloacae*

**Fig 2:**
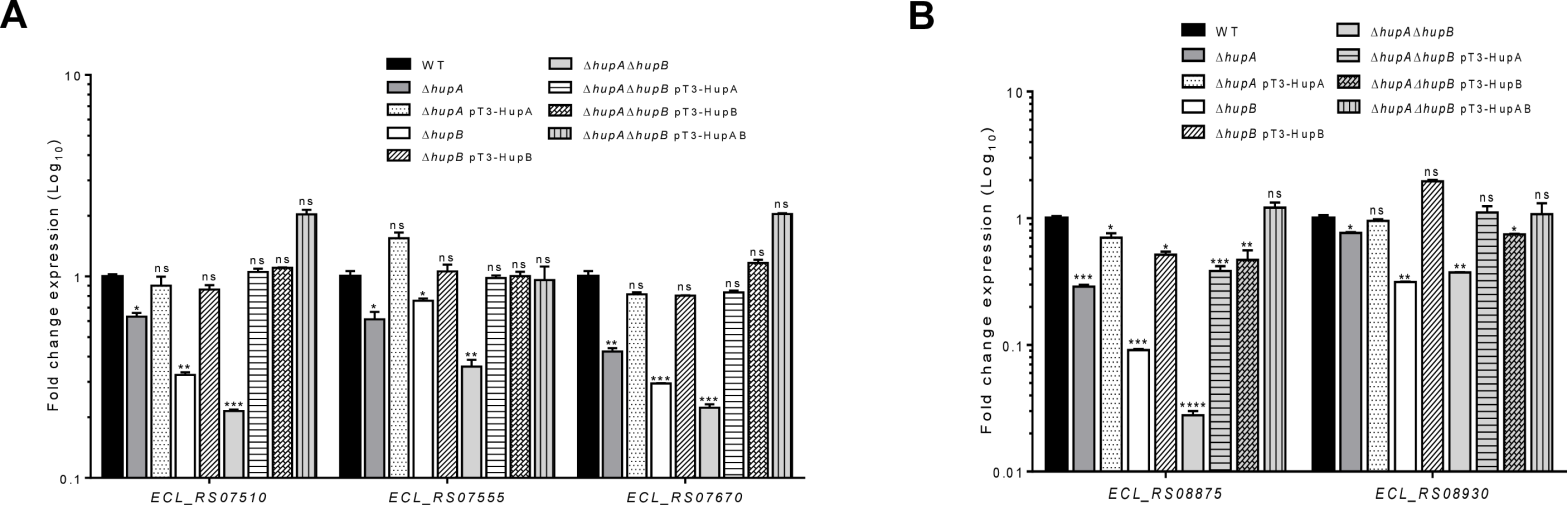
*E. cloacae* T6SS-1 and T6SS-2 are positively regulated by HU. Fold change expression (RT-qPCR) of the first genes of putative operons belonging to T6SS-1 in TSB (**A**) and T6SS-2 in DMEM (**B**). WT, Δ*hup* mutants and complemented Δ*hup* mutants were grown at 37°C for 6 h. 16S rRNA was used as a reference gene for normalization. Data represent the mean of three independent experiments performed in triplicates. Statistically significant in relation to the WT bacteria; ns: not significant; **: *P* < 0.01; ***: *P* < 0.001; ****: *P* < 0.0001.

HU is a heterodimeric protein consisting of two subunits, HUα and HUβ, encoded by the *hupA* and *hupB* genes, respectively (17). To assess the regulatory role of HU, the gene expression of T6SS-1 genes in TSB and T6SS-2 genes in DMEM upon 6 h of growth was evaluated by reverse transcription-quantitative polymerase chain reaction (RT-qPCR), using the Δ*hupA*, Δ*hupB*, and Δ*hupA* Δ*hupB* mutants. We first determined the mRNA levels of three different T6SS-1 genes, the first of three putative operons. The mRNA levels of *ECL_RS07510*, *ECL_RS07555*, and *ECL_RS07670* genes that encode T6SS-1 were significantly reduced in both *hup* mutants compared to the WT strain (Fig. 2A). Overall, the absence of the HupB subunit showed a more substantial defect than HupA. Moreover, the transcription of T6SS-1 genes was dramatically diminished in the *hupA hupB* double mutant, suggesting the effect that exerts the homo- and heterodimeric forms in the regulation of T6SS-1 (Fig. 2A). A similar effect was observed in the mRNA levels of T6SS-2 genes *ECL_RS08875* and *ECL_RS08930* whose expression was decreased in the *hup* mutant strains (Fig. 2B). Furthermore, the introduction of plasmids pT3-HupA, pT3-HupB, and pT3-HupAB into the corresponding Δ*hupA*, Δ*hupB*, or Δ*hupA* Δ*hupB* mutants restored the expression of both T6SS genes clusters to similar levels to those of the WT strain (Fig. 2).

### HU directly binds to the promoter regions of T6SS-1 and T6SS-2

**Fig 3:**
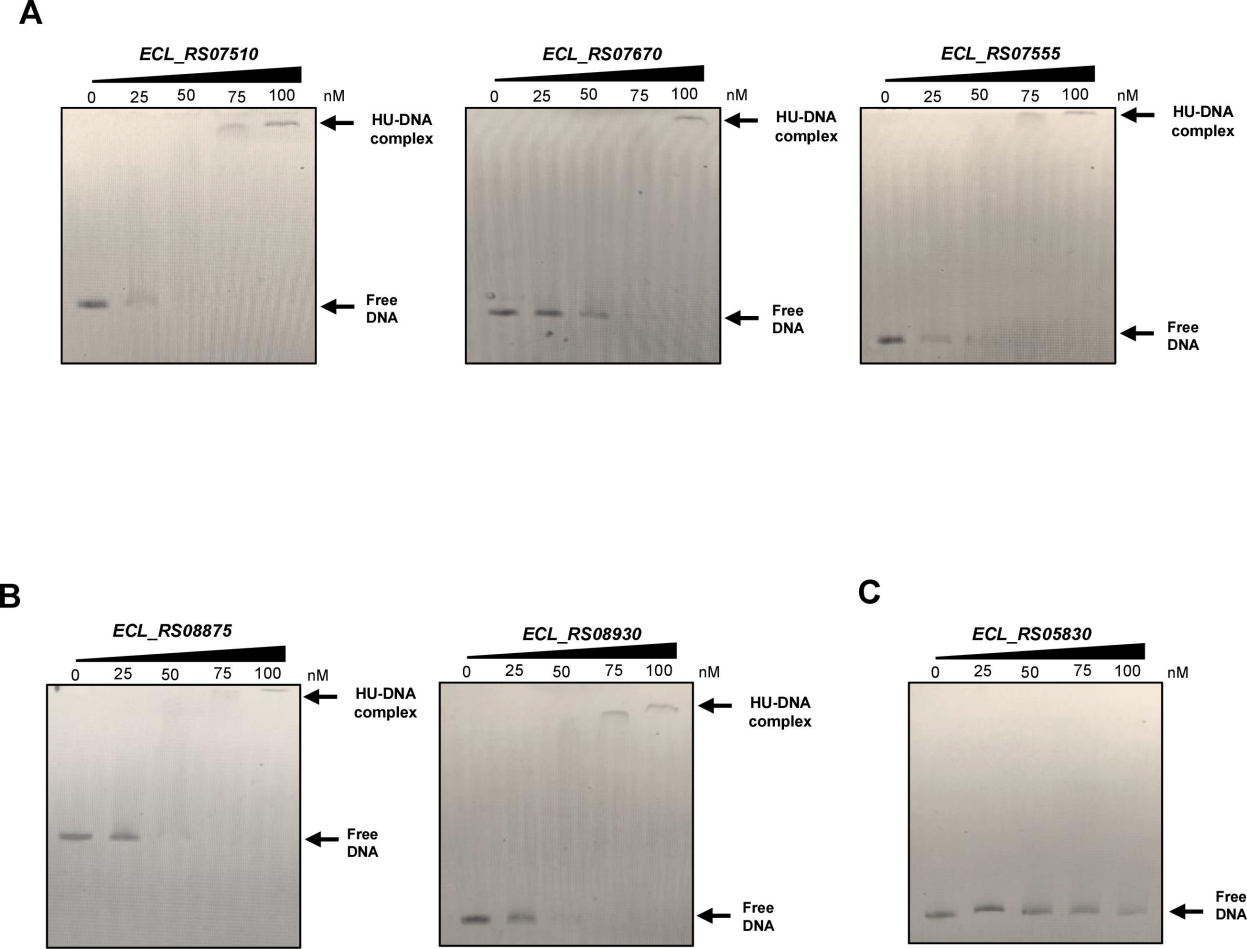
HU binds to the promoters of both T6SS-1 and T6SS-2. (**A**) *E. coli* HU binds directly to the promoter regions of the T6SS-1 genes *ECL_RS07510*, *ECL_RS07670*, and *ECL_RS07555*. (**B**) Interaction of *E. coli* HU with the promoter regions of the genes *ECL_RS08875* and *ECL_RS08930* from T6SS-2. (**C**) As a negative control, the *hupB* (*ECL_RS05830*) coding region was evaluated. Free DNA and HU-DNA complexes stained with ethidium bromide are indicated.

To determine whether HU directly regulates both T6SS in *E. cloacae*, electrophoretic mobility shift assays (EMSAs) were performed with *E. coli*-purified HU protein (97% identical to *E. cloacae* HU) and the DNA corresponding to the promoter regions of the first genes belonging to putative operons of both T6SS-1 and T6SS-2. HU bound to both the *ECL_RS07510* and *ECL_RS07670* promoter regions since the HU-DNA complex was detected at 75 and 100 nM of HU protein, respectively. Nevertheless, in the case of *ECL_RS07555*, the HU-DNA complexes were detected at 75 nM of HU (Fig. 3A). When the T6SS-2 was tested, HU bound to *ECL_RS08875* and *ECL_RS08930* promoter regions at 75 nM (Fig. 3B). As a negative control, DNA encompassing the *hupB* (*ECL_RS05830*) coding region was assessed (Fig. 3C), although HU bound to this fragment at higher concentrations of 100 nM under the tested conditions. These results show that HU directly binds to the T6SS promoters evaluated.

### Bacterial competition is turned on by HU

**Fig 4:**
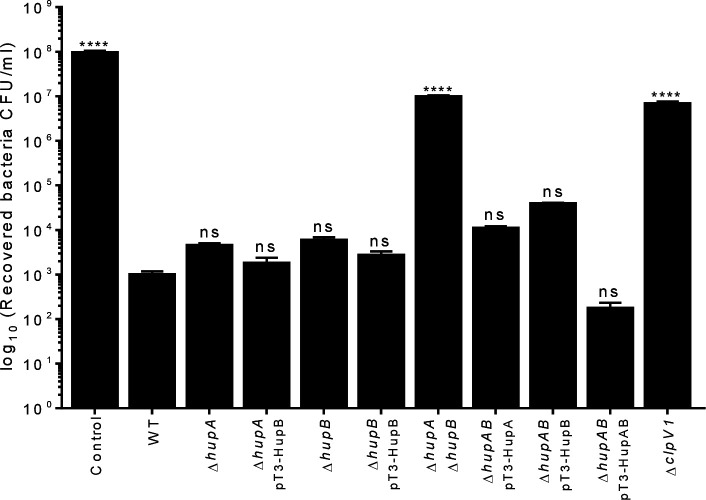
HU protein is relevant for T6SS-1-dependent bacterial competition of *E. cloacae*. Comparison of the survival of *E. coli* MC4100 against WT *E. cloacae*, Δ*hupA*, Δ*hupB*, and Δ*hupA*Δ*hupB* mutants and complemented mutant strains. Survival rates are expressed in CFU/mL. *E. coli*/LB and *E. coli*/*E. cloacae* Δ*clpV1* mixes were used as negative and positive controls, respectively. Statistically significant with respect to the WT strain; ns: not significant; ***: *P* < 0.001; ****: *P* < 0.0001.

The T6SS acts as an antibacterial weapon, killing other bacteria by injecting effector proteins, thereby helping the microorganism to compete more effectively against other bacterial species in its growth environment (32, 33). To investigate the role of HU in this T6SS-1-associated phenotype, we used the *E. coli*-carrying pMPM-T6 plasmid as a target strain in the antibacterial competition assay. The WT *E. cloacae* strain was able to kill *E. coli* (~5Log_10_); however, the absence of either subunit of HU (Δ*hupA* or Δ*hupB*) did not significantly affect *E. cloacae* bacterial competition against *E. coli* (Fig. 4). Interestingly, the absence of both subunits of HU drastically reduced the killing activity *E. cloacae* against the prey (~4Log_10_) (Fig. 4). The antibacterial activity of the Δ*hupA* Δ*hupB* double mutant was restored to WT levels by the introduction of a plasmid that express either one or both HU subunits. A more substantial bactericidal effect was noted when both subunits were expressed (Fig. 4). These results demonstrate that the HU protein turns on bacterial competition, which is T6SS-1-dependent in *E. cloacae*.

### HU is required for biofilm formation and adherence to epithelial cells

**Fig 5:**
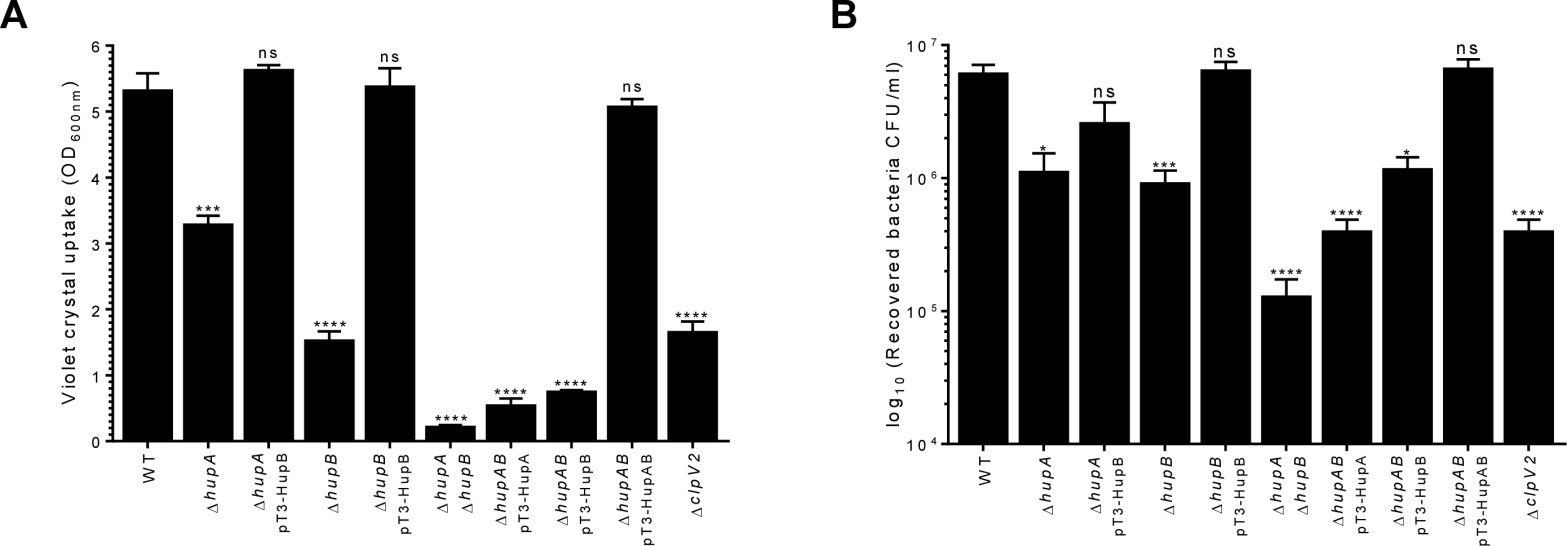
Role of HU on *E. cloacae* biofilm formation and cell attachment. (**A**) Quantification of biofilm formation by the CV protocol. WT *E. cloacae,* Δ*hup* mutants and complemented Δ*hup* mutants were grown 24 h in DMEM and biofilm detected as described in the methods section. (**B**) Adherence of WT *E. cloacae*, Δ*hup* mutants and complemented mutant strain backgrounds, after 2 h of infection in HeLa cell monolayers. *E. cloacae* Δ*clpV2* was used as a positive control for both biofilm formation and cell adherence. Statistically significant differences between WT *E. cloacae* and their respective HU isogenic mutants; ns: not significant; **: *P* < 0.01; ***: *P* < 0.001; ****: *P* < 0.0001.

In *E. cloacae*, our group showed that the adherence to epithelial cells and biofilm formation are T6SS-2 traits associated with virulence (12). To explore if HU regulates both phenotypes, which are T6SS-2-dependent, we evaluated the ability of *E. cloacae* WT, *hup* mutants, and the complemented mutant strains to produce biofilm and adhere to epithelial cells. With respect to biofilm formation, the Δ*hupA* and Δ*hupB* mutant strains showed a decrease of 40% and 70%, respectively, compared to the WT strain (Fig. 5A). Interestingly, the Δ*hupA* Δ*hupB* double mutant showed a dramatic diminishing (~25-fold) of this phenotype when it was compared to the WT strain. The Δ*hup*-complemented single mutants, which express either HupA or HupB, were able to restore the biofilm formation to WT levels (Fig. 5A). Interestingly, in the case of the complementation of Δ*hupA* Δ*hupB* double mutant, the ability to form biofilm was restored only when both HupA and HupB subunits were expressed in plasmids.

Next, the analysis of the role of HU in adherence of *E. cloacae* to HeLa epithelial cells showed a reduction of~8- and 14-fold of the Δ*hupA* and Δ*hupB* mutant strains, respectively, compared to WT strain (Fig. 5B). Like in the biofilm formation, the Δ*hupA* Δ*hupB* double mutant showed a more significant decrease (~26-fold) in the adherence to epithelial cells compared to the WT strain. When the *hup* single mutants were evaluated, the adherence to HeLa cells was restored to WT levels by the introduction of plasmids that expressed each single subunit of HU (Fig. 5B). Nevertheless, the Δ*hupA* Δ*hupB* double mutant was only able to fully restore the adhesion phenotype when this mutant was complemented with a plasmid that carries both subunits of HU (Fig. 5B). These data show that the heterodimeric HU protein is required for the biofilm formation and adherence to epithelial cells in *E. cloacae*.

### The absence of HU affects the gut colonization of *E. cloacae*

**Fig 6:**
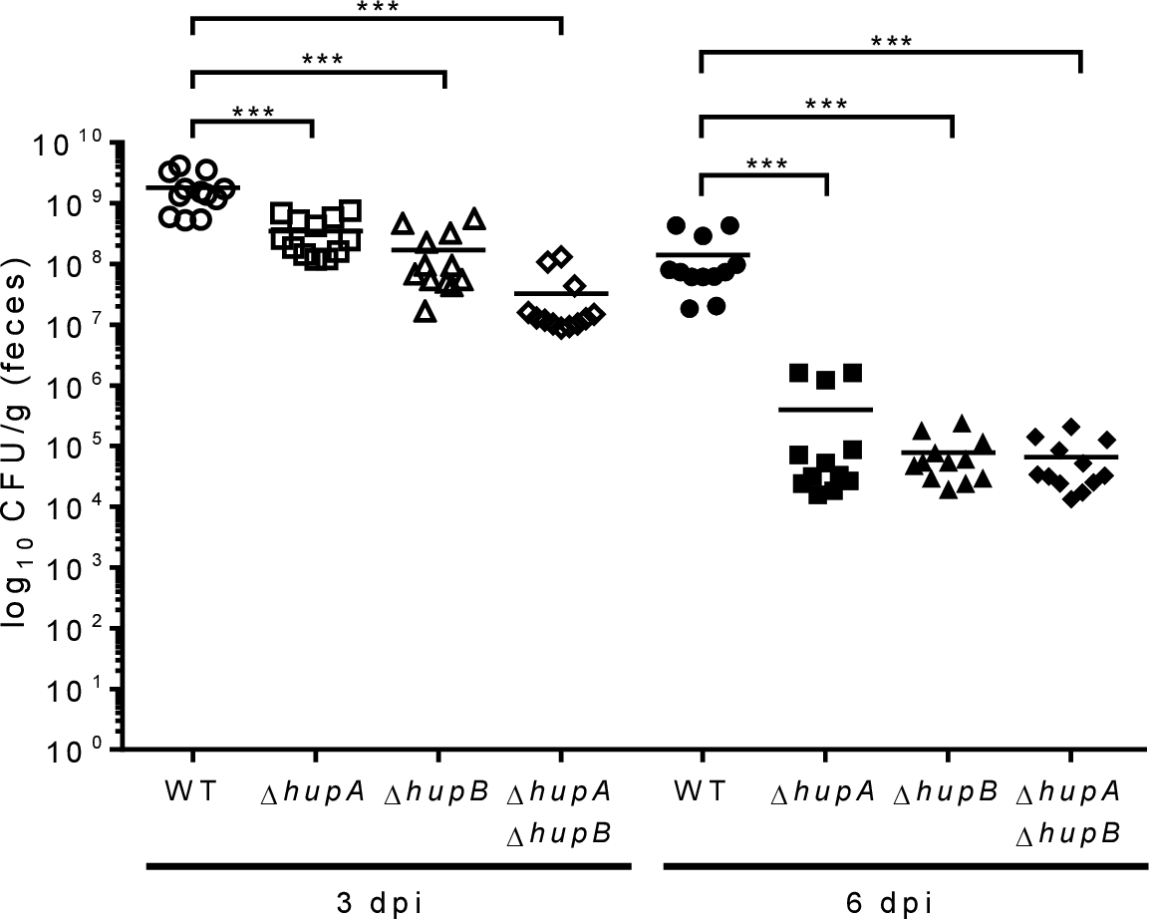
*E. cloacae* requires HU nucleoid protein during the gut colonization. BALB/c mice were infected by i.g. inoculation with 10^8^ CFU/mL with the WT strain and their respective isogenic mutants of HU subunits. The bacterial colonization was assessed after 3 and 6 d.p.i.. Statistically significant differences between WT *E. cloacae* and their respective T6SS isogenic mutants; *: *P* < 0.05; **: *P* < 0.01; ***: *P* < 0.001; ****: *P* < 0.0001.

Given that both T6SSs are associated with the bacterial pathogenesis of *E. cloacae* (12), we investigated the *in vivo* contribution of the protein HU in the colonization of the mouse gut by *E. cloacae*. BALB/c mice were infected with *E. cloacae* WT strain and the ∆*hupA*, ∆*hupB*, and Δ*hupA* Δ*hupB* isogenic mutants (Fig. 6). After 3 days post-infection (p.i.), the ∆*hupA* and ∆*hupB* single mutant strains showed a decrease in the colonization of ~8- and 15-fold, respectively, compared to the WT strain. Moreover, very low CFU numbers were recovered in the Δ*hupA* Δ*hupB* double mutant due to a reduction of ~55-, 13-, and 6-fold compared to WT, ∆*hupA*, and ∆*hupB* backgrounds, respectively (Fig. 6). On day 6 p.i., the absence of HupA or HupB dramatically affected on the CFU numbers of *E. cloacae* with a reduction on the colonization levels of ~2.5Log_10_- and 3.2Log_10_-fold of the ∆*hupA* and ∆*hupB* single mutants, respectively, compared to the WT strain (Fig. 6). Interestingly, the Δ*hupA* Δ*hupB* double mutant showed similar values of reduced colonization (~3.4Log_10_) as the Δ*hupB* mutant (Fig. 6), suggesting a main role of the HupB subunit. These data strongly suggest that both subunits of HU (HUα and HUβ) are required to promote the expression of both T6SSs during intestinal colonization of *E. cloacae*.

## DISCUSSION

The T6SS is a powerful weapon used by many Gram-negative bacteria for a variety of functions, including inter-bacterial competition and virulence (34, 35). The regulation of T6SS expression in bacterial pathogens is crucial to understanding the functioning of these systems in causing host infectious diseases and maintaining competitive advantages in polymicrobial communities. *E. cloacae* strain 13047 is an opportunistic human pathogen with two T6SSs, which are not expressed under the same synthetic growth media, suggesting independent functions for the multiple ecological niches that *E. cloacae* may encounter during its life cycle (12). Nevertheless, the transcriptional regulators regulating the T6SSs activity in *E. cloacae* still need to be clarified. This study provides evidence that both *E. cloacae* T6SS gene clusters are positively regulated by the histone-like protein HU. The NAP HU is one of the most abundant proteins in *E. coli*, and it has been suggested to play an essential role in bacterial nucleoid organization and transcriptional regulation (21).

Our results showed that the deletion of either *hupA* or *hupB* resulted in a significant reduction in the expression of the first genes of putative operons encoding T6SS-1 in TSB and the T6SS-2 in DMEM, indicating that HU positively regulates gene expression in both T6SS. Likewise, in *S*. Typhimurium and *Vibrio parahaemolyticus*, HU activates the expression of genes that encode the type III secretion system (T3SS), another needle-like nanomachine required for bacterial virulence (25, 27). In addition, the nucleoid protein HU acts as a positive regulator of the cholera toxin by promoting CTXφ prophage secretion (36). Here, by EMSA, we demonstrated that *E. coli* HU directly binds to the promoter regions on both T6SS-1 and T6SS-2 gene clusters in *E. cloacae*. We hypothesize that HU could either act as a classic transcriptional activator recruiting the RNA polymerase by direct interaction or alter the local topology of the promoter region, allowing access to the transcription machinery. Albeit HU was found to positively regulate the *F. tularensis* T6SS genes (28), to our knowledge, this is the first study in which the major histone-like protein HU has been described as an activator on T6SS genes by direct binding to the DNA and subsequently the T6SSs-associated phenotypes such as bacterial competition, cell adherence, biofilm formation, and intestinal colonization.

In Gram-negative pathogens such as *Vibrio fluvialis*, *V. parahaemolyticus*, *Aeromonas hydrophila*, *Pseudomonas aeruginosa*, and *S.* Typhimurium, the bactericidal ability associated with the T6SS is controlled by global transcriptional regulators (IHF, H-NS, and Fur) or two-component systems (FleS/FleR) (37–41). The analysis of how HU activates the T6SS-1 clearly showed that the lack of both genes, which encode the HUα and HUβ subunits, impaired the antibacterial competition of *E. cloacae* against *E. coli*. In contrast, the T6SS-2 in *E. cloacae* plays an essential role in forming biofilm and adherence to eukaryotic cells (38). In this study, the HU protein activated both the biofilm formation and cell adherence of *E. cloacae*. The deletion of *hupA* or *hupB* genes reduced these T6SS-2-associated phenotypes of *E. cloacae*. However, the reduction levels in both phenotypes observed in the Δ*hupA* Δ*hupB* double mutant were greater than the Δ*clpV2* mutant (that reflects a non-functional T6SS-2), suggesting that in addition to T6SS-2, HU protein regulates other virulence determinants that *E. cloacae* expresses during the adherence to both abiotic and biotic surfaces. In summary, HU plays a critical role in the assembly and function of T6SSs and other uncharacterized virulence factors in *E. cloacae.*

Several reports indicate that the T6SS is required for virulence in many pathogenic bacteria (32, 42–44). Here, we demonstrated that HU is also required for the *E. cloacae* intestinal colonization of BALB/c mice. The absence of either Hup subunit showed reduced levels compared to the WT strain. However, the absence of both genes ∆*hupA* and ∆*hupB* had a higher effect in colonization, suggesting that both subunits of HU are essential in gut colonization, first, competing against other bacteria found in the intestinal microbiota, and, second, allowing the adherence of *E. cloacae* to epithelial cells.

Moreover, our findings indicate that the absence of HU impacts the growth of *E. cloacae* in TSB and DMEM at 37°C. This growth defect is likely due to the deregulation of many genes, including some related to bacterial growth regulation. Unlike bacteria from the *Enterobacteriaceae* and *Vibrionaceae* families, Gram-positive and Gram-negative pathogens such as *Helicobacter pylori*, *F. tularensis*, *Porphyromonas gingivalis*, *Xanthomonas citri*, *Streptococcus intermedius*, and *Mycobacterium tuberculosis*, the HU protein functions solely as a homodimer formed by HupB subunits, as *the hupA* gene (which encodes the HupA subunit) is absent in these bacteria. In this context, only the overexpression of HupB in the Δ*hupA* Δ*hupB* double mutant fully restored *E. cloacae’s* growth, supporting the dominant role of HUβ observed in genetic expression, biofilm formation, and gut colonization.

In conclusion, our findings identify a previously unrecognized role of HU in promoting inter-bacterial competition, host cell adhesion, biofilm formation, and outstandingly, in intestinal colonization in mice for *E. cloacae* by direct positive regulation of both T6SSs. Therefore, the positive regulation of the expression of T6SS-1 and T6SS-2 by HU represents an increase in the adaptability of *E. cloacae* to different niches and hosts as part of their pathogenesis scheme.

## MATERIALS AND METHODS

### Bacterial strains and culture conditions

Bacterial strains and plasmids used in this study are listed in Table 1. Bacterial cultures were routinely grown in 250-mL flasks containing 50 mL of lysogeny broth (LB) or DMEM with high glucose (4.5 g/L). An initial inoculum of OD_600_ of 0.05 was incubated at 37°C in a shaking incubator at 200 rpm. When necessary, media were supplemented with antibiotics: ampicillin (200 µg/mL), kanamycin (50 µg/mL), chloramphenicol (34 µg/mL), and tetracycline (10 µg/mL).

**TABLE 1:** Bacterial strains and plasmids used in this study

Strain or plasmid	Description	Reference
Strains		
*E. cloacae* WT	WT *E. cloacae* strain ATCC 13047	ATCC
*E. cloacae* ∆*hupA*	*E. cloacae* ∆*hupA*::Km^R^	This study
*E. cloacae* ∆*hupB*	*E. cloacae* ∆*hupB*::Km^R^	This study
*E. cloacae* ∆*hupA* ∆*hupB*	*E. cloacae* ∆*hupA*::Km^R^ ∆*hupB*::Cm^R^	This study
*E. cloacae* ∆*clpV1*	*E. cloacae* ∆*clpV1*::FRT	(12)
*E. cloacae* ∆*clpV2*	*E. cloacae* ∆*clpV2*::FRT	(12)
MC4100	Cloning strain	(45)
BE257*recA*	C600 *leu, pro, lac, tonA, str, recA*	(46)
Plasmids		
pMPM-T3	p15A derivative low-copy-number cloning vector, *lac* promoter; Tc^R^	(47)
pT3-HupA	pMPMT3 derivative expressing HupA subunit from the *lac* promoter	This study
pT3-HupB	pMPMT3 derivative expressing HupB subunit from the *lac* promoter	This study
pT3-HupAB	pMPMT3 derivative expressing HupA and HupB subunits from the *lac* promoter	This study
pRLM118	*E. coli hupA* and *hupB* genes are expressed from PL promoter	(46)
pMPM-T6	p15A derivative cloning vector, pBAD (*ara*) promoter; Tc^R^, Sp^R^	(47)
pKD119	pINT-ts derivative containing the λ Red recombinase system under an arabinose-inducible promoter; Tc^R^	(48)
pKD4	pANTsy derivative template plasmid containing the kanamycin cassette for λ Red recombination; Ap^R^	(48)
pKD3	pANTsγ derivative template plasmid containing the chloramphenicol cassette for λ Red recombination; Ap^R^	(48)

### Construction of *E. cloacae* mutants

*E. cloacae* ATCC 13047 was targeted for mutagenesis of *hupA* and *hupB* genes, following the procedure previously reported (48) with some modifications. Each purified PCR product was electroporated into competent *E. cloacae* carrying the lambda-Red recombinase helper plasmid pKD119, whose expression was induced by adding L-(+)-arabinose (Sigma) at a final concentration of 1.0%. PCR fragments containing *hupA* and *hupB* sequences flanking a kanamycin cassette were generated using gene-specific primer pairs (Table 2), and the pKD4 plasmid was used as a template. For the Δ*hupA* Δ*hupB* double mutant, we amplified a PCR fragment containing *hupB* sequence flanking a chloramphenicol cassette using the pKD3 plasmid as a template. PCR and sequencing confirmed the respective mutations.

**TABLE 2:** Primers used in this study^*a*^

Primer	Sequence (5′−3′)	Target gene
For qPCR		
ECL_RS07510-RT-5′	ACGCTTGTCACCGGTAAAAC	*ECL_RS07510*
ECL_RS07510-RT-3′	TTGATTACCGCACGCATTGG	
ECL_RS07555- RT-5′	TTGCTGTGGTGGATTTGTCG	*hcp1*
ECL_RS07555- RT-3′	ACACCGGCTGGACTGATATTAC	
ECL_RS07670- RT-5′	CGCATCGATTTCACGGTTATCC	*vgrG1*
ECL_RS07670- RT-3′	TTCACGCGGCCATATTTGTC	
ECL_RS08875- RT-5′	AATGTGACGCTGCGCTTTTC	*impB2*
ECL_RS08875- RT-3′	AATTACGCATCGCCAGCATG	
ECL_RS08930- RT-5′	TCCCGGGATTAACAGCCTTTC	*ECL_RS08930*
ECL_RS08930- RT-3′	TTGCTGCTCCGTTTTCACTG	
ECL_rrsH- RT-5′	CAGCCACACTGGAACTGAGA	*rrsH*
ECL_rrsH- RT-3′	GTTAGCCGGTGCTTCTTCTG	
For mutagenesis		
Ecl-hupA-H1P1	TATAAAGAGAGGAAGAGAACAGTGAATAAATCTCAACTGATTGATGTAGGCTGGAGCTGCTTCG	*hupA*
Ecl-hupA-H2P2	AACTGTTCACTGCCACGCGTCTTACTTAAC TGCGTCTTTCAGTGCATATGAATATCCTCCTTAG	
Ecl-hupB-H1P1	TATAAAGAGAGGAAGAGAACAGTGAATAAATCTCAACTGATTGATGTAGGCTGGAGCTGCTTCG	*hupB*
Ecl-hupB-H2P2	TTCCCTGAAACGGGAAAGCAATCAGTTTACTGCGTCTTTCAGCGCATATGAATATCCTCCTTAG	
For mutant characterization		
Ecl-hupA-F	TTTGGCATTTTCGTCGCAC	*hupA*
Ecl-hupA-R	AATGACAAAAGGGGCGTTG	
Ecl-hupB-F	GTCATGGCAGGCCTGATATAAC	*hupB*
Ecl-hupB-R	CGCACAAATCAGGTCTGGAC	
For constructions		
hupA-XhoI-5′	CAC*CTCGAG*AGGATAACTTATGAACAAGACTCAACT	*hupA*
hupA-EcoRI-3′	TCA*GAATTC*CGCGTCTTACTTAACTGCGTC	
hupB-XhoI-5′	TAT*CTCGAG*AGGAAGAGAACAGTGAATAAATCTCA	*hupB*
hupB-EcoRI-3′	GAA*GAATTC*AAGCAATCAGTTTACTGCGTCT	
hupAB-TD-3′	GTTGAGATTTATTCACTGTTCTCTTCCTTTACTTAACTGCGTCTTTCAGTG	*hupA*
hupAB-TD-5′	CTGAAAGACGCAGTTAAGTAAAGGAAGAGAACAGTGAATAAATCTCAAC	*hupB*
For EMSA		
ECL_RS07510-5′	TAATTCAGGGCGGAAAAGTC	*ECL_RS07510*
ECL_RS07510-3′	CGCGCATTTTCATATGCTTTTCC	
ECL_RS07555-5′	TTTTGTGAGTTTCGCCCGG	*hcp1*
ECL_RS07555-3′	GCCATAATATCTACTCTTCGTGG	
ECL_RS07670-5′	GGGAGGTTTCCTTATGTTTCGG	*vgrG1*
ECL_RS07670-3′	GGAGCTGAACGGTAATTCGG	
ECL_RS08875-5′	GTGAAGATGGCGCTGCATTG	*impB2*
ECL_RS08875-3′	AGCCATAGCAGTCCCTTTCC	
ECL_RS08930-5′	GTGACGAAGAGAAAGGTTGATTGG	*ECL_RS08930*
ECL_RS08930-3′	GGTCTGGGGTAGCGTTCTG	
ECL_hupB-5′	TAT*CTCGAG*AGGAAGAGAACAGTGAATAAATCTCA	*hupB*
ECL_hupB-3′	GAA*GAATTC*AAGCAATCAGTTTACTGCGTCT	

^*a*^ Italic letters indicate the respective restriction enzyme site in the primer. The sequence corresponding to the template plasmid pKD4 or pKD3 is underlined.

### Construction of plasmids

The pT3-HupA, pT3-HupB, and pT3-HupAB plasmids were generated by cloning *hupA* and *hupB* genes of *E. cloacae*, respectively, into the pMPM-T3 plasmid (see primers in Table 2). The PCR products were digested with XhoI and EcoRI enzymes. Then, the digested PCR products were ligated into the pMPM-T3 vector, which was also previously digested with the same restriction enzymes. The identities of the inserts were confirmed by DNA sequencing.

### Quantitative RT-PCR

The hot phenol method was used to extract total RNA (49). Residual DNA was removed with a TURBO DNA-Free Kit (Ambion, Inc.), and the NanoDrop ONE (Thermo Scientific) and a bleach denaturing 1.5% agarose gel were assessed for evaluating the quantity and quality of RNA, respectively (50). To synthesize cDNA, 1 µg of RNA, 5 pmol/µL of random hexamer primers, and 20 U/µL of RevertAid M-MulV-RT (Thermo Scientific) were used. Primer3Plus software (http://www.bioinformatics.nl/cgi-bin/primer3plus/primer3plus.cgi/) was used to design specific primers listed in Table 2. A LightCycler 480 instrument (Roche) was used to quantify gene expression levels by qPCR. Nucleic acid amplification was determined in triplicate three independent experiments. In each set of reactions, the *rrsH* gene, which encodes 16S rRNA, was used as a reference gene to normalize the cDNA amount. The absence of contaminating DNA was tested by the lack of amplification products after 45 qPCR cycles using RNA as template. In addition, qPCR control reactions with no RNA template and with no reverse transcriptase enzyme were run in all experiments. The relative gene expression was calculated using the 2^-ΔΔCt^ method (51).

### Purification of the HU protein

*E. coli* strain BE257*recA* (C600 *leu*, *pro*, *lac*, *tonA*, *str*, and *recA*) harboring the plasmid pRLM118 (PL promoter drives the transcription of *hupA* and *hupB* genes) was used to overexpress the *E. coli* HU protein (97% identical to *E. cloacae* HU). The purification of *E. coli* HU protein was described previously (46). A 20% SDS-PAGE and Lowry assay (Bio-Rad) were used to confirm the HU protein purity and the concentration, respectively.

### Electrophoretic mobility shift assays

To evaluate HU binding to the promotor sequence, DNA probes containing the intergenic regulatory region of the first genes belonging to operons of the T6SS-1 and T6SS-2 of *E. cloacae* were amplified by PCR with primer pairs enlisted in Table 2. A region of *hupB* (*ECL_RS05830*) was amplified by PCR with primers ECL_hupB-5′ and ECL_hupB-3′ and used as a negative control. PCR products were purified using the QIAquick PCR Purification Kit (Qiagen). Proteins and DNA fragments were mixed in 1× binding buffer (10× buffer: 400 mM HEPES, 80 mM MgCl_2_, 500 mM KCl, 10 mM dithiothreitol, 0.5% NP-40, and 1 mg/mL bovine serum albumin) (52) to a final volume of 20 mL and incubated at room temperature for 30 min. DNA fragments were resolved by electrophoresis in 6% non-denaturing polyacrylamide gels using 0.5× Tris-borate-EDTA buffer. The DNA bands were stained with ethidium bromide and visualized under UV light.

### Bacterial competition

Experiments were performed as previously described (32), with some modifications. The *E. cloacae* and *E. coli* strains were grown overnight with aeration in 5 mL of LB containing the appropriate antibiotics. From the overnight culture, subcultures were performed in TSB medium and incubated at 37°C with constant shaking until reaching an OD_600_ of ~1.0, and they were mixed in a 1:4 ratio (predator:prey). Aliquots of 20 µL of the mixed bacterial culture were spotted onto LB agar and incubated at 37°C for 2 h. The bacterial spot on the agar surface was subsequently removed and vigorously resuspended in PBS, and the CFUs per milliliter of surviving prey strains were measured by plating serial dilutions on solid selective media. The selective medium contained 100 µg/mL of spectinomycin for prey strains previously transformed with pMPM-T6 plasmid. The output/input ratio of the prey-to-predator strains was interpreted as survival and included at least three independent assays.

### Biofilm formation assay on abiotic surface

Bacterial adhesion to the abiotic surface (polystyrene) was analyzed using 96-well plates (53). Overnight cultures of bacteria grown in LB (10 µL) were added to 1 mL of DMEM. This volume was distributed in quintuples (100 µL per well) into a 96-well plate and incubated at room temperature for 24 h. Unbound bacteria were removed from the wells after washing the cultures three times with PBS, and bound bacteria were stained with 1% crystal violet (CV) and incubated for 20 min at room temperature. After incubation, the wells were rinsed thrice with PBS, and the dye was solubilized in 100 µL of 70% ethanol. Lastly, the amount of extracted CV was determined by measuring the OD_595_ in an ELISA Multiskan Plate Reader (Thermo Scientific). These experiments were performed in triplicate at three independent times.

### Bacterial adherence

Monolayers of the HeLa (ATCC CCL-2) cell line (7 × 105 cells/well) were infected with the indicated strains from an LB overnight culture at a multiplicity of infection of 100. Epithelial cells were grown in DMEM with 10% fetal bovine serum (FBS). After infection, eukaryotic cells were incubated in DMEM with no FBS for 1 h at 37°C under an atmosphere of 5% CO_2_. After 1 h of incubation, cells were washed thrice with PBS and then lysed with a solution of 0.1% Triton X-100 for 15 min. After homogenization, the lysates containing total cell-associated bacteria were diluted serially in PBS and plated onto LB agar plates to enumerate adherent bacteria. The results are the mean of at least three experiments performed in triplicate on different days.

### Mouse inoculation experiments

Mice infection experiments were performed using the BALB/c strain. Mice groups (*n* = 5) were pretreated with 50 mg of streptomycin 24 h before infection with *E. cloacae* strains. Mice were infected by intragastric (i.g.) inoculation with 1 × 10^8^ CFU/mL of bacteria under sterile conditions. Fresh fecal pellets were collected directly into microtubes at 3 and 6 days post-infection (d.p.i.). Pellets were resuspended vigorously in sterile PBS 1×, and CFUs per gram of feces were determined by plating serial dilutions on LB agar plates with ampicillin (200 µg/mL).

### Statistical analysis

All data are means from three independent experiments. Statistical analysis was performed using Prism 8.0 software (GraphPad, Inc., San Diego, CA, USA). A one-way analysis of variance was performed, followed by Tukey’s multiple-comparison test and unpaired Student’s *t*-test. *P* values of ≤ 0.05 were considered statistically significant.

## Data Availability

The authors confirm that the data supporting the findings of this study are available within the article.
